# Application of artificial intelligence-based dual-modality analysis combining fundus photography and optical coherence tomography in diabetic retinopathy screening in a community hospital

**DOI:** 10.1186/s12938-022-01018-2

**Published:** 2022-07-20

**Authors:** Rui Liu, Qingchen Li, Feiping Xu, Shasha Wang, Jie He, Yiting Cao, Fei Shi, Xinjian Chen, Jili Chen

**Affiliations:** 1 Department of Ophthalmology, Shanghai Jing’an District Shibei Hospital, 4500, Gonghexin Road, Jing’an, Shanghai, 200443 China; 2 Department of Ophthalmology and Vision Science, Eye and ENT Hospital, Fudan University, Shanghai, 200031 China; 3 Key Laboratory of Myopia of State Health Ministry, and Key Laboratory of Visual Impairment and Restoration of Shanghai, Shanghai, 200031 China; 4 Laboratory of Myopia, Chinese Academy of Medical Sciences, Shanghai, 200031 China; 5 School of Electronic and Information Engineering, Soochow University, Suzhou, 215006 Jiangsu China; 6 Suzhou Big Vision Medical Imaging Technology Co. Ltd., Suzhou, 215000 Jiangsu China

**Keywords:** Artificial intelligence, Deep learning, Diabetic retinopathy, Optical coherence tomography

## Abstract

**Background:**

To assess the feasibility and clinical utility of artificial intelligence (AI)-based screening for diabetic retinopathy (DR) and macular edema (ME) by combining fundus photos and optical coherence tomography (OCT) images in a community hospital.

**Methods:**

Fundus photos and OCT images were taken for 600 diabetic patients in a community hospital. Ophthalmologists graded these fundus photos according to the International Clinical Diabetic Retinopathy (ICDR) Severity Scale as the ground truth. Two existing trained AI models were used to automatically classify the fundus images into DR grades according to ICDR, and to detect concomitant ME from OCT images, respectively. The criteria for referral were DR grades 2–4 and/or the presence of ME. The sensitivity and specificity of AI grading were evaluated. The number of referable DR cases confirmed by ophthalmologists and AI was calculated, respectively.

**Results:**

DR was detected in 81 (13.5%) participants by ophthalmologists and in 94 (15.6%) by AI, and 45 (7.5%) and 53 (8.8%) participants were diagnosed with referable DR by ophthalmologists and by AI, respectively. The sensitivity, specificity and area under the curve (AUC) of AI for detecting DR were 91.67%, 96.92% and 0.944, respectively. For detecting referable DR, the sensitivity, specificity and AUC of AI were 97.78%, 98.38% and 0.981, respectively. ME was detected from OCT images in 49 (8.2%) participants by ophthalmologists and in 57 (9.5%) by AI, and the sensitivity, specificity and AUC of AI were 91.30%, 97.46% and 0.944, respectively. When combining fundus photos and OCT images, the number of referrals identified by ophthalmologists increased from 45 to 75 and from 53 to 85 by AI.

**Conclusion:**

AI-based DR screening has high sensitivity and specificity and may feasibly improve the referral rate of community DR.

## Background

Diabetic retinopathy (DR), one of the most common complications of diabetes mellitus, is an eye disease known to cause moderate-to-severe visual loss and is the leading cause of blindness in working-age people suffering from long-standing diabetes [1–3]. Very often, the disease does not show overt symptoms until it reaches an advanced stage. However, a regular follow-up can allow early detection and treatment of vision-threatening retinopathy, which enables the prevention of up to 98% of visual loss due to DR [4, 5]. Given that most vision loss from DR is avoidable through early detection and effective treatment strategies [6, 7], many national and international societies have long endorsed screening for DR [8], which is most commonly in the form of point-of-care ophthalmoscopy by trained ophthalmologists or retinal photography with either local interpretation or telemedicine-based screening programs with centralized grading [9].

In recent years, with the improvement of computer processing speed, artificial intelligence (AI) programs have better assisted in the diagnosis and management of clinical diseases, especially in the field of ophthalmology. The application of this technology in ophthalmology is currently focused mainly on diseases with a high incidence, such as DR and age-related macular degeneration (ARMD) [10]. Several current studies have proposed AI screening algorithms for DR, which show high accuracy, sensitivity and specificity [11–22]. We recently applied AI-based DR screening in community hospitals and achieved high sensitivity and specificity in detecting DR and referable diabetic retinopathy (RDR) [23]. However, in the process of screening, we also found some deficiencies, especially in the identification of diabetic macular edema (DME). As a diagnosis of DME requires identification of macular thickening, screening for DME using non-stereoscopic fundus photographs is likely to cause errors. Therefore, if optical coherence tomography (OCT), as the primary tool for macular disease detection, can be introduced as a screening test in addition to fundus photography, the sensitivity to detect fundus diseases will increase further. To the best of our knowledge, the simultaneous use of fundus photography and OCT for the screening of DR has not been reported before. Herein, we applied deep learning-based AI grading of DR based on both fundus photography and OCT to community hospital clinics. This study aims to assess the accuracy of AI-based screening for DR by fundus photos combined with OCT images and to explore the feasibility and application value of this dual-modality for DR screening in a community hospital.

## Results

**Fig. 1:**
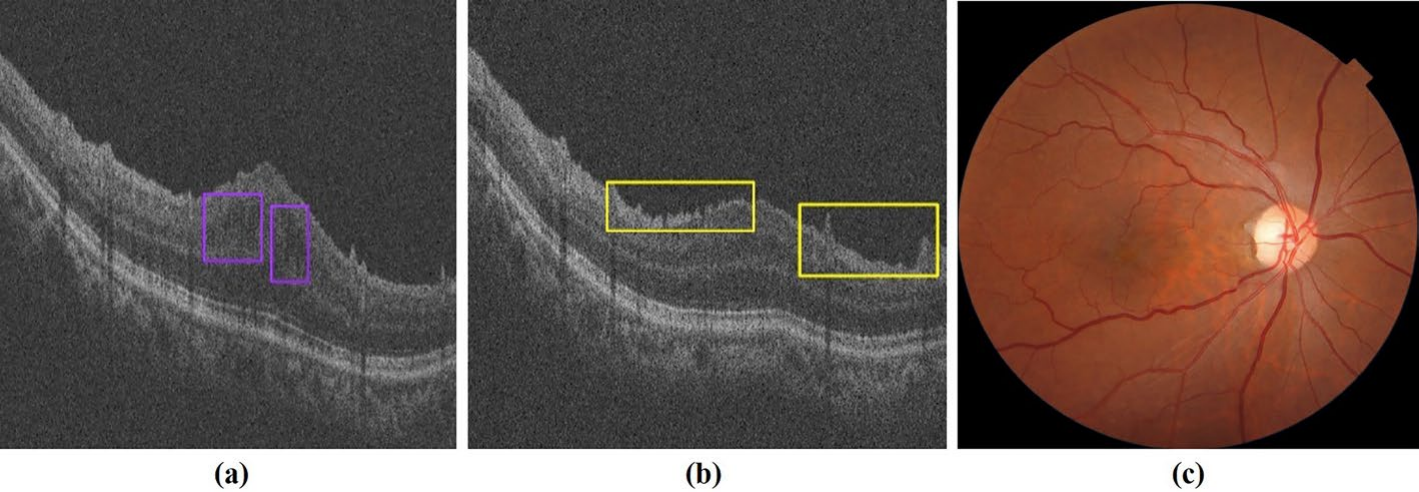
OCT-Fundus-AI diagnosis results. **a** OCT B-scan with detection of retinal fluid (purple bounding box). **b** OCT B-scan with detection of the epiretinal membrane (yellow bounding box). **c** No obvious abnormalities on the fundus

**Fig. 2:**
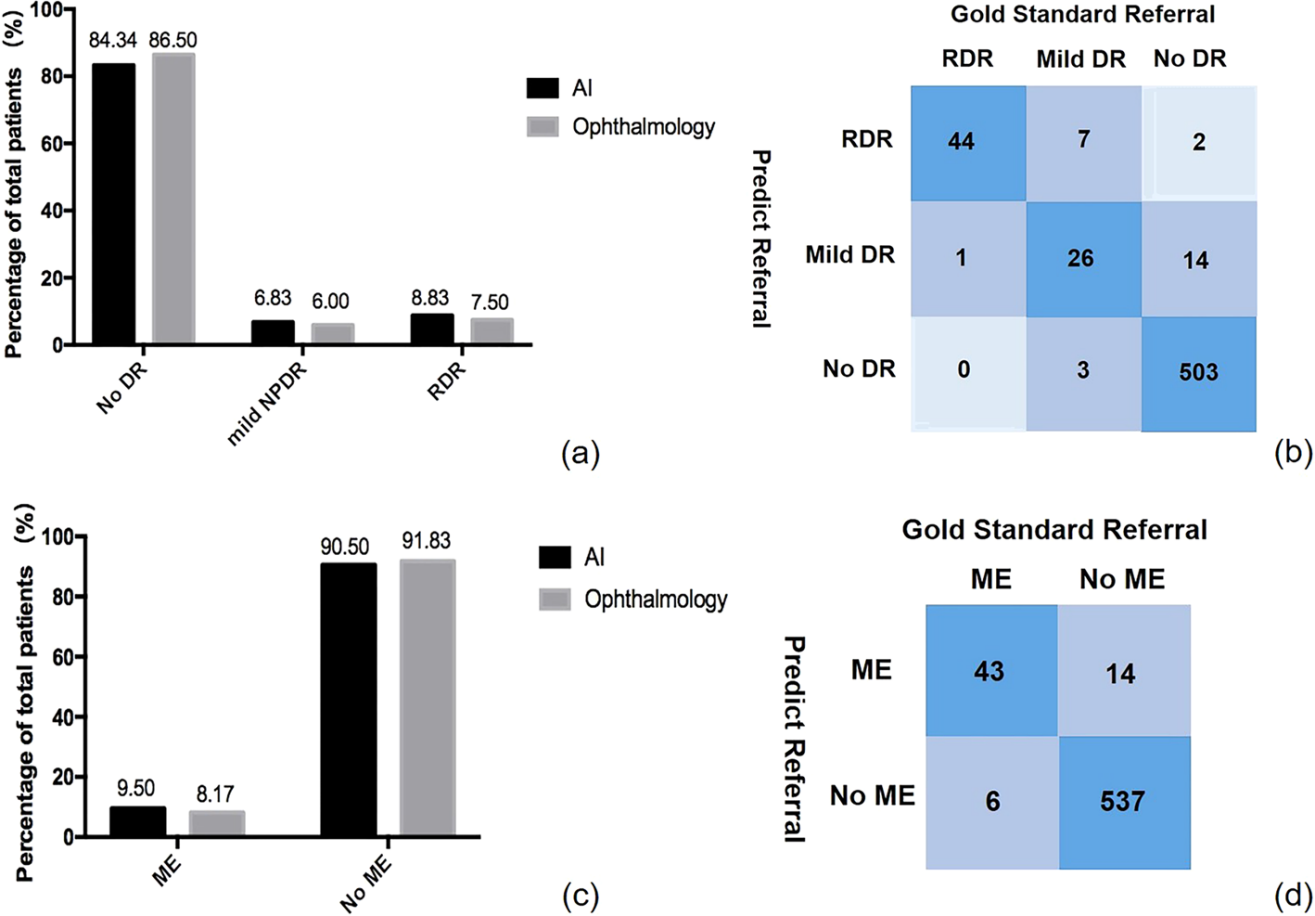
**a** Comparison of diabetic retinopathy (DR) grading between ophthalmologists and AI. **b** The confusion matrix for the DR detection. **c** Comparison of macular edema (ME) classifications between ophthalmologists and AI. **d** The confusion matrix for the ME detection

Six hundred diabetic patients participated in the DR screening, including 324 men and 276 women, and for each person, a random eye was taken for testing. The average age of the participants was 67.26 ± 7.02 years. For fundus photography, 1200 retinal images were obtained and graded in this study. According to the International Clinical Diabetic Retinopathy (ICDR) classification scale, 78 participants with DR were detected by both ophthalmologists and AI. Ophthalmologists detected DR in 81 (13.5%) participants, while AI detected DR in 94 (15.6%) participants. Figure 1 shows an OCT-Fundus-AI diagnostic result compared with those of single modality-based diagnosis systems, which indicates the superiority of the multi-modality-based retinal disease diagnostic system. Most of the participants' fundus photographs revealed no DR. RDR was diagnosed in 45 (7.5%) participants based on manual grading and in 53 (8.8%) participants using AI (Fig. 2a). The confusion matrix (Fig. 2b) shows that 27 incorrect cases (23 due to over referral and 4 due to under referral). For DR detection, the sensitivity and specificity achieved by AI were 91.67% (95% CI 77.5–98.2) and 96.92% (95% CI 95.0–98.2), the PPV (positive predictive value) were 82.98% (95% CI 73.5–89.7) and NPV (negative predictive value) were 99.41% (95% CI 98.1–99.8), respectively. For RDR detection, the sensitivity and specificity achieved by AI were 97.78% (95% CI 88.2–99.9) and 98.38% (95% CI 96.9–99.3), the PPV were 83.02% (95% CI 69.7–91.5) and NPV were 99.82% (95% CI 98.8–99.9), respectively. The area under the curve (AUC) was 0.944 (95% CI 0.922–0.962) when testing the ability of AI to detect DR; for the detection of RDR, the AUC was 0.981 (95% CI 0.966–0.990).

**Fig. 3:**
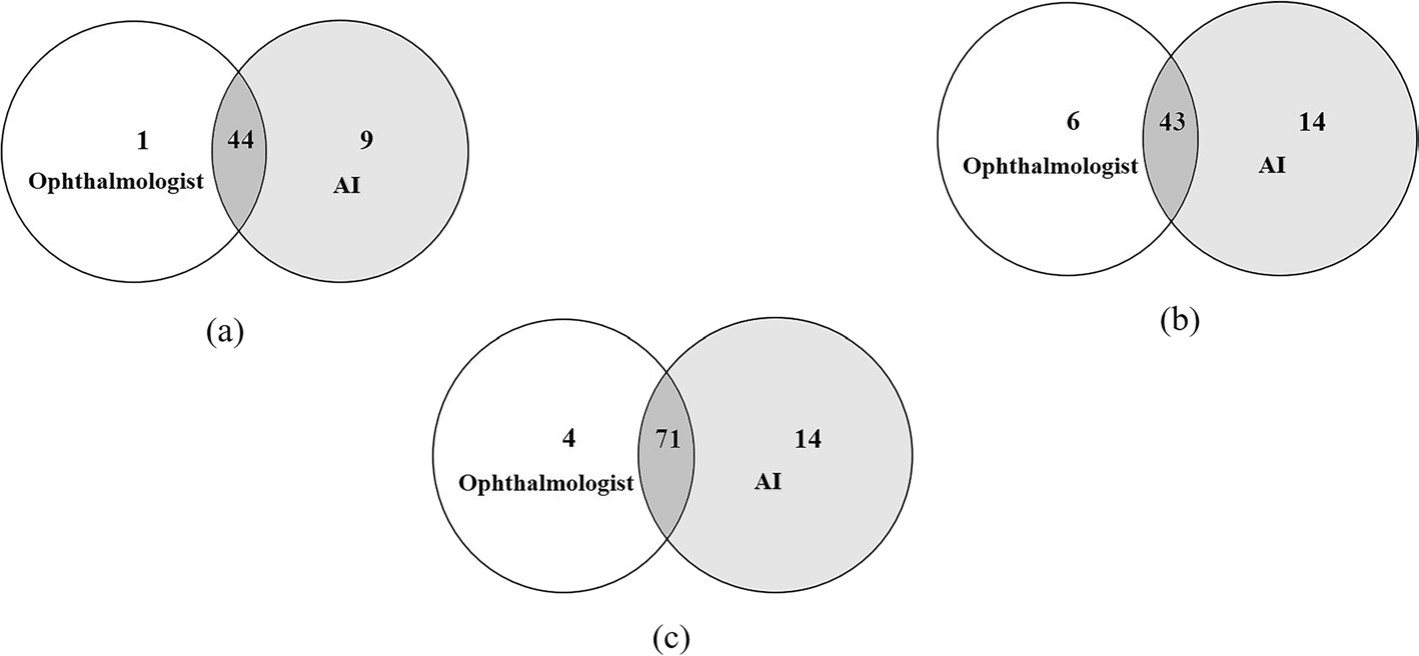
Venn diagram showing the overlap comparison of the number of referrals between human and automated grading: **a** fundus photography, **b** OCT, and **c** fundus photography combined with OCT

For OCT images, 43 participants with ME were detected by both ophthalmologists and AI, 20 of whom were considered to have DME. ME was detected by ophthalmologists in 49 (8.2%) participants and in 57 (9.5%) participants by AI (Fig. 2c). The confusion matrix (Fig. 2d) shows that 20 incorrect cases (14 due to over referral and 6 due to under referral). For ME detection, the sensitivity, specificity and AUC of AI were 91.30% (95% CI 72.0–98.9), 97.46% (95% CI 95.8–98.6) and 0.944 (95% CI 0.922–0.962), the PPV were 75.44% (95% CI 62.0–85.5) and NPV were 98.9% (95% CI 97.5–99.6), respectively. A matched diagnosis of RDR between ophthalmologists and AI grading was observed in 44 participants by fundus photography. However, when combined with OCT, the number of referrals given by ophthalmologists increased from 45 to 75, where the increase included 7 for DME and 23 for other reasons. For AI, the number of referrals increased from 53 to 85. The number of referrals jointly identified by ophthalmologists and AI also increased to 71, as shown in Fig. 3 and Table 1.

**Table 1:** Sensitivity, specificity and AUC of AI for detection of different degrees of DR and ME with ophthalmologist grading as reference standard

Stage	Sensitive, %	(95% CI)	Specificity, %	(95% CI)	AUC	(95% CI)	*P* value
Any DR	91.67	77.5–98.2	96.62	95.0–98.2	0.944	0.922–0.962	< 0.0001
RDR	97.78	88.2–99.9	98.38	96.9–99.3	0.981	0.966–0.990	< 0.0001
ME	91.30	72.0–98.9	97.46	95.8–98.6	0.944	0.922–0.962	< 0.0001

## Discussion

The AI system used in this study can achieve relatively good results after a short period of training and learning using a small batch of data. In the past 5 years, the sensitivity and specificity of the AI-assisted DR screening system based on color fundus photographs reported by various working groups around the world were 87–99% and 91.4–99.0%, respectively, and the AUC was 0.989–0.991 [11–19]. Our results showed that the sensitivity of DR screening using AI was 91.67%, the specificity was 96.92% and the AUC was 0.944, which are similar to those in previous studies and may therefore meet the needs of clinical screening. Moreover, the sensitivity of detecting RDR in our study was 97.78%, and the AUC was 0.981 [17], which are close to the results of the former study, where the authors demonstrated the performance of a deep learning enhancement algorithm used for automatic RDR detection. The sensitivity of their algorithm was 96.8%, and the AUC was 0.980. Moreover, the specificity of our results was much higher, reaching 98.38% compared with 87%. Additionally, the sensitivity for RDR detection in our study was much higher than that for DR detection, indicating that more advanced disease corresponds to more accurate identification by our system.

DME is the most common cause of visual loss in those with diabetic retinopathy and is increasing in prevalence globally [24–26]. The prevalence of DME in patients with diabetic retinopathy is 2.7–11% and depends on the type of diabetes and the duration of the disease, but for both types 1 and 2, after 25 years of duration, the prevalence is approximately 30% [24]. In DME, the macula is thickened due to increased extracellular fluid derived from hyperpermeable retinal capillaries, affecting detailed central vision. Notably, DME can occur at any stage of DR, whether non-proliferative diabetic retinopathy (NPDR) or proliferative diabetic retinopathy (PDR) [27]. However, before the use of OCT, the detection of macular edema in clinical studies was performed with 2-dimensional (2-D) non-stereoscopic digital fundus photography. Without stereopsis, monocular fundus photography studies identified DME using surrogate markers of thickening, such as lipids near the foveal center, macular focal/grid laser scars, or localized color changes in the macula [28–30]. Therefore, affected by pupil size and refractive media, screening for DME using non-stereoscopic fundus photographs is likely to have a very high false-positive rate (e.g., > 86% in Hong Kong [31] and > 79% in the UK [32]). Recent studies have focused on demonstrating that deep learning algorithms can be trained using OCT images to detect DME and other retinal diseases. Kermany et al. [33] first applied deep learning and transfer learning techniques in the detection of ARMD and DME from 2-D OCT images. Their models achieved high performance (sensitivity ≥ 96% and specificity ≥ 94%). The latest study is from Wang et al. [34] who applied a deep learning model with an adapted feature pyramid network to detect 15 categories of retinal pathologies from OCT images as common signs of various retinal diseases. Their results also reached a high level with a sensitivity ≥ 94% and specificity ≥ 98%.

Our statistical analysis showed that the sensitivity of ME screening using AI was 91.30%, the specificity was 97.46%, and the AUC was 0.944, which are slightly lower than those of previously reported cases, possibly because our study was a real-world study conducted in community hospitals. The subjects were generally older, and some subjects had cataracts and other diseases at the same time, which may affect the quality of some pictures. When fundus photography was combined with OCT, the number of referrals increased from 45 to 75, including 7 for DME and 23 for other reasons. Although we detected only 7 patients with DME in this screening, retinal thickness abnormalities caused by other causes should not be ignored, and reliable referral can also allow these patients to receive treatment as soon as possible. Thus, OCT appears to provide ophthalmologists with a critical reference for clinical diagnosis. It can reduce not only the rate of missed diagnosis but also inappropriate referral caused by false-positives.

The advantage of this study is that our algorithm can process and analyze fundus photography and OCT at the same time. Our study and previous studies have shown that AI-based DR screening for outpatients seems to be feasible. After the examination, AI will issue the report on-site without waiting. The doctors in the community hospital can address the report according to the condition of the patient and select the appropriate patients for referral to an ophthalmologist at a superior hospital. During the examination, none of the patients had mydriasis, which was more easily accepted.

Currently, most AI-assisted screening for DR has been carried out worldwide in the form of simple fundus photography. Based on this practice, we applied innovations and proposed for the first time that OCT can improve the screening rate and accuracy, detect patients with early DME, and increase the number of effective referrals.

Although our research has achieved good results, several limitations of this study must be considered. First, the datasets used in this study were collected from only one community hospital, and the subjects were generally elderly and could not cover all age groups. Second, the sample size was relatively small, resulting in an uneven distribution of patients with different grades of retinopathy.

## Conclusion

In this study, both AI-assisted DR screening systems based on color fundus photographs and AI-assisted ME screening systems based on OCT have high sensitivity and specificity. This system can be feasibly implemented in the outpatient clinic of a community hospital, and more patients requiring referral can be identified to improve the referral rate of community DR.

## Methods

### Participants

This study was performed following the tenets of the Declaration of Helsinki. Diabetes patients who attended Pengpu Town Community Hospital of Jing’an district, Shanghai, were invited to participate in this study. Patients with diagnosed diabetes were included, and patients with systemic diseases except diabetes that affects the retina or those who underwent intraocular surgery were excluded from this study. All subjects were 18 years of age or older, and written informed consent was obtained from each subject. This study was approved by the ethical committee of Shibei Hospital, Jing’an District, Shanghai (ChiCTR1900024528).

### Datasets

All the images in this study were acquired using the Topcon 3-dimensional OCT-1 Maestro (Topcon, Tokyo, Japan), which could acquire both color fundus and OCT images. For fundus photography, 45° color retinal photographs were taken for each eye. Retinal images with two fields, macula-centered and disc-centered, were captured according to the EURODIAB protocol [35]. For OCT, 50,000 axial scans were captured per second, producing a 20-μm lateral and 6-μm axial resolution. All the OCT images had a field of view of 6 mm × 6 mm. AI equipment was installed and used in the community hospital. Images of each participant were immediately analyzed by AI and transmitted to two ophthalmologists simultaneously. Patient information was anonymized and unconnected to OCT images before being transferred to the investigators. All the included images could be accurately diagnosed by retinal specialists, and images that were unclear because of hazy media, such as serious cataracts, fixation failure during image capture, severe motion or shadow artifacts, and other reasons, were excluded.

### Ground truth

All fundus photographs and OCT images were graded independently by two ophthalmologists (retina specialists, Kappa (κ) = 0.844, 0.864) who were masked to each other and AI device outputs. In the grading process, ophthalmologists did not have access to obtain the fundus photography and OCT images at the same time. When the results between the two retina specialists were inconsistent, a third retina specialist made a final decision. All three experts had more than ten years of experience. The grading of retinopathy was evaluated according to the ICDR [8]. The diagnostic criteria for ME in fundus photography was the presence of a typical stellate configuration, i.e., radially orientated perifoveal cysts [36]. OCT images of those patients were obtained to identify whether there was concomitant ME. There were three OCT patterns of DME, which included the DRT as sponge-like retinal swelling of the macula with reduced intraretinal reflectivity, the CME as intraretinal cystoid spaces of low reflectivity and highly reflective septa separating cystoid-like cavities in the macular area, and the SRD as a shallow elevation of the retina and an optically clear space between the neurosensory retina and retinal pigment epithelium [37]. The criteria for referral were DR grades 2 (moderate NPDR), 3 (severe NPDR), and 4 (PDR)and/or the presence of ME [38].

### Automated grading

**Fig. 4:**
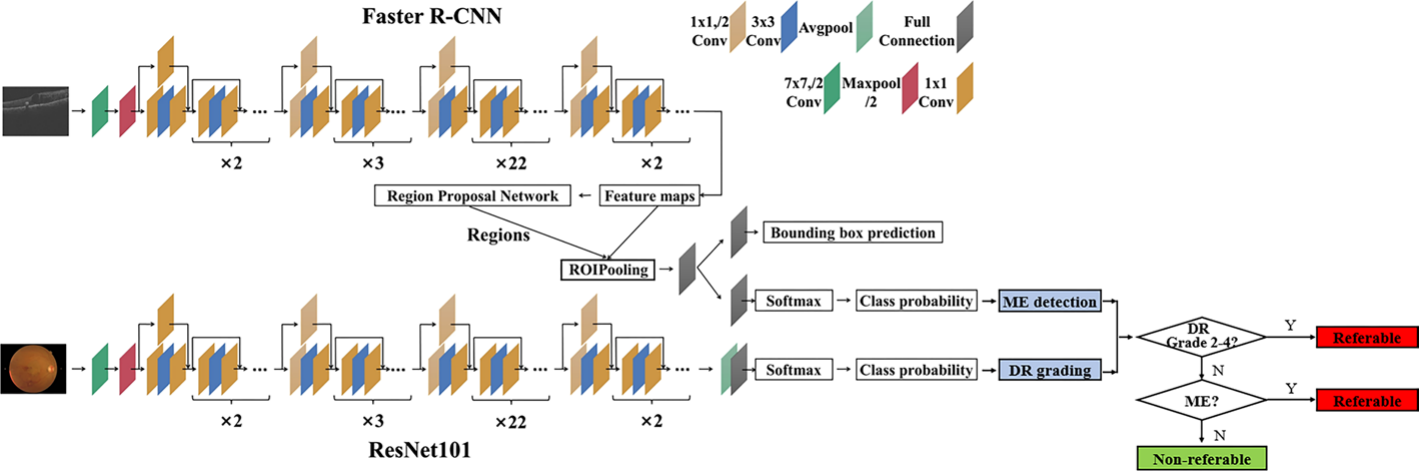
Flowchart of AI-based dual-modality DR screening algorithm

The AI device performed automated analysis and identified signs from both retinal photographs and OCT images with AI software (Bigvision Inc., Suzhou, China). Then, a DR screening report including referral recommendations was generated and delivered to the participant immediately. This multimodality retinal disease diagnosis system applied deep learning algorithms to fundus images and OCT scans to achieve disease classification and detection and integrated the results to generate a comprehensive screening report with referral recommendations. The specific deep learning framework used was ResNet 101 [39] for DR stage classification on fundus images and Faster R-CNN [40] for retinal abnormality detection on OCT scans. Figure 4 shows the flowchart of our algorithm.

For fundus photography, the classification network of our proposed diagnostic system was based on ResNet 101, which was initialized by the pre-trained model trained on the ImageNet dataset. Since DR lesions such as micro-aneurysms appeared as a small region, down sampling of the input fundus image might cause the lesion regions to disappear. Therefore, the fundus images were resized to 1024 × 1024, this resolution strongly ensured that the small lesion regions were not eliminated by down-sampling preprocessing. A large-scale dataset was collected for the training of the DR classification network, which contained more than 50,000 labeled fundus images from multiple centers. The images were labeled by ophthalmologists as DR grade 0–4.

**Fig. 5:**
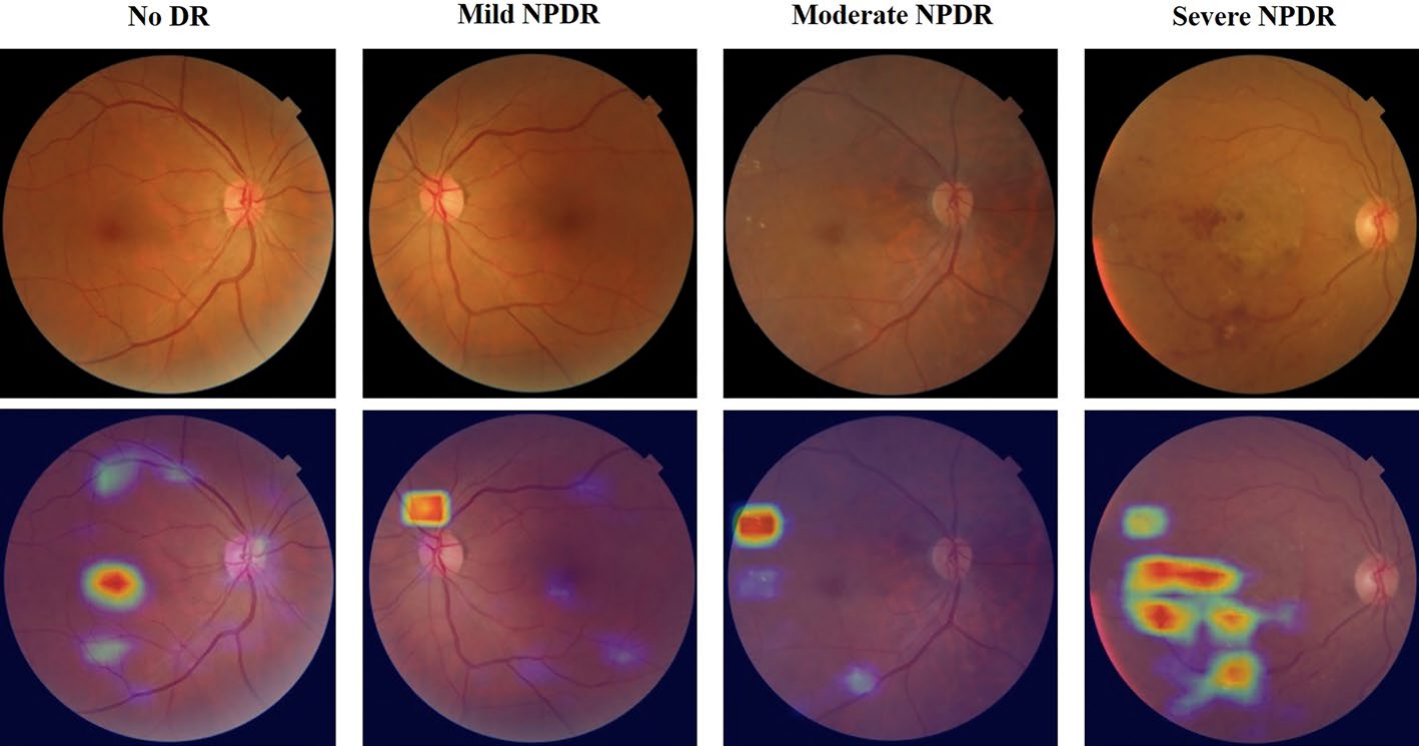
Examples of classification heatmaps of different levels of DR

The DR grading model was validated on a test set containing 1200 fundus images. The overall accuracy was 0.7825, and the sensitivity and specificity for detecting referable DR (grade 2–4) was 0.7172 and 0.9242, respectively. Figure 5 shows that the AI software yields DR stage classification results based on the values of the output vector from the heatmap.

For OCT, the detection network was built based on Faster R-CNN, where the ResNet 101 architecture was used as the backbone model for feature extraction from input OCT B-scans. The training dataset contained more than 20,000 OCT B-scans from more than 2000 patients from multiple centers, involving 16 types of common retinal abnormalities. The images were labeled by ophthalmologists, who drew a bounding box containing each pathological area, and gave a label specifying its type. The input images were resized according to the rules specified by Faster R-CNN [40], and the resulting short side ranges from 800 to 1333 pixels. The output predicted bounding boxes of lesions and class labels were further filtered based on 3D spatial context information from the volumetric scan.

**Fig. 6:**
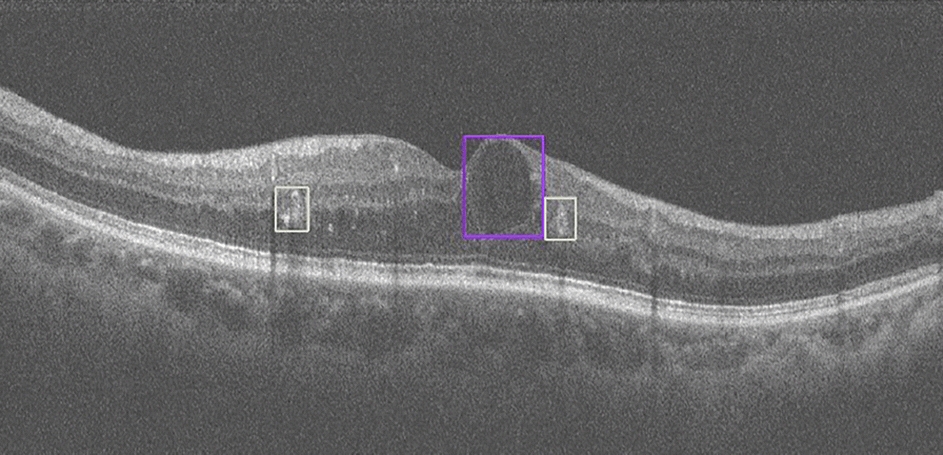
Retinal abnormality detection results. Detected retinal exudates (white bounding box) and retinal fluid (purple bounding box)

The abnormality detection model was validated on a test set containing 3247 OCT B-scans. The overall accuracy is 0.9743, and the mean sensitivity and specificity is 0.9142 and 0.9820, respectively. Figure 6 shows retinal abnormalities detection results from OCT B-scans by our OCT-AI diagnostic system.

Our classification and detection networks were implemented by the PyTorch framework and trained on a deep learning server with eight GeForce GTX 1080ti graphical processing units. The classification network was initialized by the pre-trained model on the ImageNet dataset. Then the Adam optimizer was used for training with 5,800,000 steps, where the initial learning rate was 4e−7. To improve the generalization abilities of the network, the training data were augmented with random flipping, cropping and rotation. For the detection network, the stochastic gradient descent optimizer was used for training with 180,000 steps, where the initial learning rate was 0.0025 and the weight decay was 0.0001. The training data were augmented by random flipping, cropping, and noise addition. The trained model has not been adjusted and is directly applied to clinical data.

### Statistical analysis

Statistical analysis of the data was performed using the SPSS Statistics 26.0 for Windows (SPSS Inc., Chicago, IL, USA). The consistency between two ophthalmologists was evaluated using the Kappa coefficient, and the sensitivity and specificity of AI automatic grading were calculated regarding the results from ophthalmologists as the gold standard. The number of referrals confirmed by AI and the ophthalmologists was calculated and compared.

## Data Availability

The datasets used and/or analyzed during the current study available from the corresponding author on reasonable request.

## Abbreviations

AI: Artificial intelligence
DR: Diabetic retinopathy
OCT: Optical coherence tomography
ME: Macular edema
ICDR: International clinical diabetic retinopathy
AUC: Area under the curve
ARMD: Age-related macular degeneration
RDR: Referable diabetic retinopathy
DME: Diabetic macular edema
PPV: Positive predictive value
NPV: Negative predictive value
2-D: 2-Dimensional
